# Measuring the impact and distress of health problems from the individual's perspective: development of the Perceived Impact of Problem Profile (PIPP)

**DOI:** 10.1186/1477-7525-4-36

**Published:** 2006-06-29

**Authors:** Julie F Pallant, RoseAnne Misajon, Elizabeth Bennett, Lenore Manderson

**Affiliations:** 1 Faculty of Life and Social Sciences, Swinburne University of Technology, P.O. Box 218, Hawthorn, Victoria 3122, Australia; 2 School of Psychology, Psychiatry & Psychological Medicine, Monash University, 900 Dandenong Rd, Caulfield East, Victoria 3145, Australia; 3 School of Population Health, The University of Melbourne, Victoria 3010, Australia

## Abstract

**Background:**

The aim of this study was to develop and conduct preliminary validation of the Perceived Impact of Problem Profile (PIPP). Based on the biopsychosocial model of health and functioning, the PIPP was intended as a generic research and clinical measurement tool to assess the impact and distress of health conditions from the individuals' perspective. The ICF classification system was used to guide the structure of the PIPP with subscales included to assess impact on self-care, mobility, participation, relationships and psychological well-being. While the ICF focuses on the classification of objective health and health related status, the PIPP broadens this focus to address the individuals' subjective experience of their health condition.

**Methods:**

An item pool of 23 items assessing both impact and distress on five key domains was generated. These were administered to 169 adults with mobility impairment. Rasch analysis using RUMM2020 was conducted to assess the psychometric properties of each set of items. Preliminary construct validation of the PIPP was performed using the EQ5D.

**Results:**

For both the Impact and Distress scales of the PIPP, the five subscales (Self-care, Mobility, Participation, Relationships, and Psychological Well-being) showed adequate psychometric properties, demonstrating fit to the Rasch model. All subscales showed adequate person separation reliability and no evidence of differential item functioning for sex, age, educational level or rural vs urban residence. Preliminary validity testing using the EQ5D items provided support for the subscales.

**Conclusion:**

This preliminary study, using a sample of adults with mobility impairment, provides support for the psychometric properties of the PIPP as a potential clinical and research measurement tool. The PIPP provides a brief, but comprehensive means to assess the key ICF components, focusing on the individuals' perspective of the impact and distress caused by their health condition. Further validation of its use across different health conditions and varying cultural settings is required.

## Background

There exist a number of models that provide a conceptualization of health and disability. Within the traditional medical model, disability is viewed as a characteristic of an individual that is caused by the health condition, requiring the need for medical intervention by professionals to manage the condition. The social model, in contrast, does not view disability as being a characteristic of an individual, but as a socially induced problem [1]. The social model differentiates impairment and disability, with the term *impairment *referring to the physical condition and *disability *to the societal and environmental barriers inhibiting social participation of individuals with impairment. In 2000, the WHO introduced The International Classification of Functioning, Disability and Health (ICF) [1], which integrates these two models and adopts a biopsychosocial approach which views biological, psychological, and social processes as integral to and interactive with physical health and illness.

Within the framework of the ICF, disability and functioning are seen as the result of complex interactions between health conditions and contextual factors, both environmental and personal. Environmental factors, both physical (climate, terrain, architecture) and social (legal and social structures, cultural and social attitudes), and personal factors, such as gender, age, education, character, coping styles and past experience, may all contribute to the impact of a specific health condition on an individual. Coupled with the shift in emphasis on the social construction of disability, there has been a growing awareness of the need to measure the impact of a health condition from the individual's perspective, taking into account social context and personal factors.

While an objective assessment of an individual's symptoms and his or her functional status is important, it only provides part of the picture. At a practical level, the biopsychosocial model considers that understanding the person's subjective experience is essential for accurate diagnosis, health outcomes and appropriate care. Hewlett [2] has argued that difficulty in performing valued activities would determine the personal impact of a particular impairment, and consequently that it was necessary to assess the perceived importance to the individual of specific activities (eg. tasks involved in dressing and grooming, preparing and eating food, personal hygiene). These importance ratings were then used to weight the individual's disability scores on the Health Assessment Questionnaire, resulting in a personal impact score (PI HAQ) [2]. The results of this study suggest that individuals with similar impairment levels (as determined by the HAQ) can have very different levels of personal impact arising from the impairment.

Other researchers have taken the individual or patient focused approach to measurement a step further. 'Patient generated outcome measures' [3] or individualized questionnaires are increasingly popular in treatment outcome studies. These measures do not consist of predefined domains (eg. self-care, physical function, social interaction) but instead, individuals are asked to nominate areas or activities in their own lives that are affected by their health condition. Typically weightings are applied to scores, based on the relative importance assigned by the individual. Examples of this type of measure include the Patient Generated Index (PGI [4]), Schedule for the Evaluation of Individual Quality of Life (SEIQOL [5,6]), and the Asthma Quality of Life Questionnaire (AQLQ [7]). While these individual or patient-generated measures provide insight into individual experience, recent reviews of such instruments have identified potential problems and disadvantages [3,8].

Many patient-generated measures use weightings to assign importance to specific domains of individual's lives, but the mathematical appropriateness of weightings in quality of life measures has recently been questioned. Trauer and MacKinnon [8], for example, show that multiplicative composites have a number of undesirable psychometric properties, making them unsuitable for many statistical procedures, although they are also problematic for practical reasons [3]. The measures have been criticized as time consuming and cumbersome to complete, resulting in missing data, and they require complex judgement tasks beyond the comprehension of some respondents with limited education or cognitive impairment. In addition, lack of standardization means that comparisons across individuals with different health conditions or across time points for the same individual are not possible. Patel et al. [3] concludes that they are not suitable for use in clinical trials or treatment evaluation, but may be better suited in the consultative process to design a therapy plan.

### Current study

In this paper, we report on the development and preliminary validation of an instrument designed to overcome some of the weaknesses of patient-generated measures, while still focusing on the impact of health condition from the individual's perspective. The instrument was designed to be generic rather than disease specific, making it suitable for use across different groups. Unlike existing measures of functioning, the instrument was not designed to assess an individual's ability to perform certain tasks or activities, but rather to better understand the impact and distress caused by the health condition. This was intended to supplement more objective measures (eg. SF-36 [9] and the Health Assessment Questionnaire [10]), providing additional information incorporating contextual factors (environment and personal) identified in the ICF. This information can play an important role in optimising the provision of health care services and setting of priorities. For example, two patients with similar health problems (eg. restricted movement in the knee joint) may experience very different levels of impact and distress. Respondent 1, a professional footballer, may experience highly elevated levels of impact and distress concerning his condition, requiring immediate treatment. In comparison, Respondent 2, a 75 year old with a sedentary life style, may report relatively little impact or distress. This understanding of the meaning of the health condition for the individual can be used in clinical settings to guide the development of treatment programs and to assess outcomes and effectiveness.

Our aim in this study was to develop a relatively short, self-report instrument to assess both the impact and the distress of health problems from the individual's perspective. A set of standardized domains was specified to allow comparison of scores across patient groups and in individuals over multiple time points. The selection of domains for inclusion was guided in part by the ICF, a review of existing measures, and a series of qualitative interviews. Items were selected to assess the impact and distress of the health condition on the Activities and Participation domains of the ICF (Chapters 4 to 9) [1]. An additional component was included to assess the impact on the individual's psychological well-being (including independence and autonomy), an area not yet well addressed in the ICF.

### Development of the Perceived Impact of Problem Profile (PIPP)

A general test plan was developed representing five separate subscales (Self-care, Mobility, Participation, Relationships, Psychological Well-being) (see Table 1 for items and corresponding ICF code). Prior to the generation of specific items, qualitative interviews were conducted to identify aspects of individuals' lives that they felt were affected by their disability and caused them distress or reduced their perceived quality of life, to ensure that all relevant aspects were identified for inclusion in the scale.

**Table 1:** PIPP items and corresponding ICF codes

**ICF Codes**	**PIPP Item Bank**	**Item no**.
**Ch 1: Mental Functions**		
b189 Mental functions other specified	Overall satisfaction with life	1
b152 Emotional functions	Moods and feelings	2
b126 Temperament and personality functions	Sense of confidence	3
*b1266 Confidence*		
**Ch 5: Self-Care**		
d510 Washing oneself	Ability to wash yourself	4
d530 Toileting	Ability to use the toilet	5
d540 Dressing	Ability to dress yourself	6
d550 Eating	Ability to feed yourself	7
**Ch 6: Domestic Life**		
d660 Assisting others	Ability to assist other members of your family	8
**Ch 4: Mobility**		
d410 Changing basic body positions	Ability to sit or stand	9
*d4101 Sitting*		
*d4104 Standing*		
d430 Lifting and carrying objects	Ability to carry things (eg bucket of water)	10
d470 Moving around using transportation	Ability to use a vehicle (eg bus)	11
d460 Moving around in different locations	Ability to move around and within your house	12
*d4600 Moving around within the home*		
d460 Moving around in different locations	Ability to move around your neighbourhood	13
*d4602 Moving around outside the home*		
**Ch 7: Interpersonal interactions & relationships**		
d740 Formal relationships	Ability to relate to people in authority (eg government officials)	14
*d7400 Relating with persons in authority*		
d750 Informal social relationships	Ability to relate to neighbours and friends	15
*d7500 Informal relationships with friends*		
*d7501 Informal relationships with neighbours*		
d760 Family relationships	Ability to relate to relatives	16
d770 Intimate relationships	Ability to have a close relationships with another person (eg husband or wife)	17
**Ch 8: Major Life Areas**		
d850 Remunerative employment	Ability to work (eg paid work, agricultural work)	21
**Ch 9 Community, social, and civic life**		
d910 Community life (d930 Religion and spirituality)	Ability to participate in community activities (eg social or religious events)	19
d920 Recreation and leisure	Ability to participate in activities you enjoy (eg sports)	20
d998 Community, social and civic life, other specified	Ability to participate in family activities (eg eating together)	18
No corresponding ICF code	Ability to live independently	22
No corresponding ICF code	Your reliance on others	23

Note. For each item respondents were asked to rate on a 6-point scale (a) 'how much impact has your current health problems had on [item of function or activity]'; and (b) 'How much distress has been caused by the impact of your health problem on [same item of function or activity] For example, for item 1 respondents were asked: Please rate how much impact your health problems had on your overall satisfaction with life. Please rate how much distress this has caused.

A total item pool of 23 items was generated representing the key domains identified (see Table 1). For each item, respondents were asked to rate on a 6-point scale (a) 'how much impact has your current health problems had on [item of function or activity]'; and (b) 'How much distress has been caused by the impact of your health problem on [same item of function or activity]'. The 6-point scale was anchored on either end by 'no impact' and 'extreme impact' for the Impact scale and by 'no distress' and 'extreme distress' for the Distress scale. For each item, an additional response option was provided for the individual to indicate that the activity was not applicable.

The wording of the items was carefully chosen to ensure that the activities were suitable for both males and females, across different age groups, and for use in different cultural contexts. This study was designed as part of a larger cross-cultural project (RESILIENCE project), with the development of items being conducted in collaboration with researchers from Malaysia and Thailand. The initial version of the PIPP was pilot tested in a series of interviews with individuals with mobility impairment in Australia, Malaysia and Thailand.

### Validation sample with locomotive disorders

Musculoskeletal conditions are the most common cause of physical disability and severe, chronic pain, affecting millions of people globally. These include a wide spectrum of conditions, including those of acute onset (e.g. trauma, injury), and short or long-term disorders (e.g. arthritis, multiple sclerosis). Due to its frequency and chronicity, and the degree of disability that can result, it has a pervasive impact on society. The frequency of musculoskeletal conditions increases with age, and with longer life-spans and hence, ageing populations, there will be an increased number of people with chronic disability disorders with increasing requirements to access healthcare services.

A recommended ICF core set for people with musculoskeletal conditions in acute care included items from the Activities and Participation component such as mobility, self-care, interpersonal interactions, relationships, handling stress and psychological demands [11]. In fact, mobility (walking) was found to have reached a consensus of at least 80% as an important and relevant domain among a range of health conditions, including low back pain, osteoporosis, rheumatoid arthritis, osteoarthritis, chronic ischaemic heart disease and diabetes mellitus [12].

The preliminary validation of the PIPP was conducted using a sample of people with mobility impairment. The impact of mobility impairment on life quality is multi-dimensional, affecting individuals by limitations in activity and restrictions in participation. Subsequent consequences can include reduced employment potential and income, lifestyle limitations, decreased ability to carry out self-care activities, increased dependency on others, and poorer quality of life. Hence, mobility impairment can have a profound impact on the psychosocial status of the individual.

## Methods

### Participants

Participants were recruited through co-operation with the Central Highlands General Practice Division. Employing a two staged modified cluster sampling [13,14], 30 general practitioners in the Central Highlands region were selected. Each practitioner was asked to select seven adult patients from his or her practice who, for any reason, experienced impaired mobility or problems with walking. Rather than diagnostic categories, a liberal criterion was used to identify potential participants, and reasons for mobility impairment included limb amputation (e.g. from accident, cancer or vascular disease), spinal cord injury, stroke, degenerative conditions (e.g. arthritis), and diseases affecting the central nervous system (e.g. multiple sclerosis).

While the aim was to recruit 210 across the 30 practices, initially 178 participants were recruited, who gave verbal consent to the general practitioner or practice nurse and were then contacted by a member of the research team. Twenty-one of these participants declined to proceed for various reasons including personal health (e.g. too ill to partake in the interview), and health of family member (e.g. nursing dying husband), while others were unable to keep appointments despite visits by the research team on more than two occasions. Advertisements were placed in a few local newspapers to increase the number of participants and an additional 12 participants were recruited using this approach. In total, 169 participants were recruited and interviewed in person for this study. Ethics approval for the study was granted by the Human Research Ethics committee of The University of Melbourne.

### Materials

Additional questions and scales were included in the questionnaire battery to allow an exploration of the validity of the PIPP. These included demographic questions, some details concerning their health condition, and the EQ5D. The EQ5D, developed by the EuroQol Group [15] is a brief standardized, non-disease-specific instrument to measure physical, emotional and social functioning and well-being, and has been used extensively worldwide. It assesses five dimensions – mobility, self-care, usual activities, pain/discomfort, and anxiety/depression. Within each domain, there are three possible responses: '*no problems,' 'some problems,' and 'unable to perform*.' These five dimensions of health are complemented by a 20-cm vertical visual analogue acale (EQ5D VAS) for the respondent to rate his or her health today from the 'best imaginable health state' set at 100 and 'worst imaginable health state' set at 0. The EQ5D was used with the permission of the EuroQoL Group.

### Statistical analyses

The PIPP Impact and PIPP Distress responses were subjected to Rasch analysis using the RUMM2020 software [16] to assess the psychometric properties of each set of items. For each of the individual subscales, a series of analyses was undertaken to assess overall model fit, threshold ordering, item fit, person fit, and differential item functioning. Rasch calibrated subscale scores were then exported into SPSS Version 12 for further statistical analyses to assess the validity of the subscales. The construct validity of the subscales was assessed in relation to the corresponding component of the EQ5D which was included in the questionnaire booklet. Further details of the Rasch procedures undertaken are provided in the section to follow; a detailed description and illustration of the Rasch analysis procedures is provided in Pallant and Tennant [17].

The RUMM2020 software [16] was used to assess how well the observed data fit the expectations of the Rasch measurement model [18]. The Rasch model provides a template that operationalizes the formal axioms that underpin measurement [19]. Initially the appropriateness of the response scale was assessed by inspection of the threshold values for each item. The term *threshold *refers to the point between two response categories where either response is equally probable. For a good fitting model, we would expect that, for each of the items, respondents with high levels of the attribute being measured would endorse high scoring responses, while individuals with low levels of the attribute would consistently endorse low scoring responses. This would be indicated by an ordered set of response thresholds for each of the items. One of the most common sources of item misfit concerns respondents' inconsistent use of these response options, resulting in what is referred to as *disordered thresholds*. This suggests that respondents have difficulty consistently discriminating between response options. Collapsing of categories was used to correct disordered thresholds to improve overall fit to the model.

Three overall fit statistics were considered to assess model fit. Two are item-person interaction statistics transformed to approximate a z-score, representing a standardized normal distribution. If the items and persons fit the model, we would expect to see a mean of approximately zero and a standard deviation of 1. The third fit statistic was an item-trait interaction statistic reported as a chi-square, reflecting the property of invariance across the trait. A significant chi-square indicates that the hierarchical ordering of the items varies across the trait, so compromising the required property of invariance.

Individual person-fit and item-fit statistics were also assessed, both as residuals (a summation of individual person and item deviations) and as a chi-square statistic. Residuals ± 2.5 are considered to indicate adequate fit to the model. The summed chi-square within each group provides the overall chi-square for the item, and the overall chi-square for items is summed to give the item trait-interaction statistic. Bonferroni corrections are applied to adjust the chi-square *p *value to take account of multiple testing [20]. An estimate of the internal consistency reliability is provided as a Person Separation Index (PSI), where the estimates on the logit scale for each person are used to calculate reliability. PSI values can be interpreted in the same way as Cronbach alpha coefficients, with values above .7 considered adequate [21].

Assessment of differential item functioning (DIF) was used to identify possible item bias [22]. This can occur when different groups within the sample (e.g. males and females), despite equal levels of the underlying characteristic being measured, respond in a different manner to an individual item. Two types of DIF were assessed statistically and graphically using the RUMM2020 software. Uniform DIF exists when one group shows a consistent systematic difference in its responses to an item, across the whole range of the attribute being measured. When there is non-uniformity in the group differences (e.g. it varies across levels of the attribute), then this is referred to as non-uniform DIF. Analysis of variance was conducted for each item, comparing scores across each level of the 'person factor' and across different levels of trait (referred to as class intervals). Uniform DIF is indicated by a significant main effect for the person factor (gender), while the presence of non-uniform DIF is indicated by a significant interaction effect (person factor × class interval).

## Results

Separate analyses are reported for the PIPP Impact and PIPP Distress items. For each set of items, Rasch analysis was conducted for the individual subscales of Self-care, Mobility, Participation, Relationships and Psychological Well-being.

### PIPP impact subscales

Prior to assessing the model fit of the subscales, the threshold ordering of the items was inspected. All items showed some degree of disordering, except item 9 (*sit or stand*). To overcome this problem, all items were recoded from the original 6-point scale (scored: 012345) by collapsing scores to form a simpler 3-point response scale (011112). This resulted in ordered thresholds for all items except item 14 (*authority*), which showed only minor disordering.

#### PIPP impact: self-care

The four self-care items included in the Impact scale were subjected to Rasch analysis to assess their ability to form a psychometrically sound subscale. The overall model fit statistics were non-significant, indicating no serious misfit to the model (item-trait interaction chi-square = 10.61, df = 8, p = .23). The PSI was .79, suggesting adequate person separation reliability, given the small number of items involved. The mean fit residual value for items was -.13 with a standard deviation (SD) of .20, while the mean fit residual for persons was -.39 with a SD of .76. No misfitting items were detected, with all individual item fit statistics being non significant (see Table 2). Items were found to be free of differential item functioning for sex, age, education level and rural/urban residence.

**Table 2:** Individual item fit statistics for PIPP: Impact scale items

	**Item**	**Location**	**SE**	**FitResid**	**DF**	**ChiSq**	**DF**	**Prob**
**Self-care**	4	-0.81	0.19	-0.20	71.75	5.26	2	0.072
	5	-0.08	0.20	-0.36	71.75	1.10	2	0.578
	6	-0.43	0.23	-0.03	71.75	0.42	2	0.810
	7	1.33	0.24	0.08	71.75	3.83	2	0.148
								
**Mobility**	9	-1.18	0.18	-0.19	124.81	3.10	2	0.212
	10	-0.42	0.15	-0.52	122.46	1.26	2	0.534
	11	0.25	0.14	1.82	123.24	0.33	2	0.850
	12	1.49	0.17	-0.33	124.81	6.48	2	0.039
	13	-0.14	0.14	-0.01	121.67	0.29	2	0.866
								
**Participation**	8	0.07	0.15	**2.78**	103.15	7.82	2	0.020
	19	0.84	0.15	-0.06	107.70	1.12	2	0.571
	19	0.55	0.16	-1.43	106.18	12.82	2	**0.002**
	20	-0.58	0.14	0.49	109.98	1.80	2	0.406
	21	-0.88	0.18	-1.04	65.99	3.14	2	0.208
								
**Relationships**	14	0.05	0.16	0.62	60.41	2.32	2	0.314
	15	0.42	0.20	0.45	60.41	2.76	2	0.252
	16	0.12	0.19	0.33	58.28	3.11	2	0.211
	17	-0.59	0.19	1.06	46.91	3.70	2	0.157
								
**Psychological well-being**	1	-1.01	0.19	-0.74	126.70	1.86	2	0.394
	2	0.48	0.18	0.02	126.70	0.29	2	0.863
	3	0.55	0.15	0.10	126.70	1.39	2	0.500
	22	0.34	0.14	-1.66	121.98	2.01	2	0.366
	23	-0.36	0.15	-0.73	125.91	2.88	2	0.237

Significant values are bolded.

#### PIPP impact: mobility

Rasch analysis of the five mobility items revealed no disordered thresholds (after rescoring 011112), good overall model fit (item-trait interaction chi-square = 11.45, df = 10, p = .32) and adequate person separation reliability (PSI = .75) (see Table 2). The mean fit residual value for items was .16 (SD = .95), while the mean fit residual for persons was -.36 (SD = 1.02). There were no misfitting items and no evidence of differential item functioning.

#### PIPP Impact: Participation

Initial inspection of the overall model fit statistics for the five participation items indicated some misfit to the model (item-trait interaction chi-square = 26.7, df = 10, p = .003) (see Table 2). All thresholds were ordered (after global recoding to 011112), however individual item fit statistics revealed one item (item 8: *assist other family members*) with a positive fit residual exceeding 2.5 (2.775). After removal of this item from the scale the overall fit statistics improved (item-interaction chi-square = 7.28, df = 8.0, p = .51). The mean fit residual value for items was .34 (SD = .68), while the mean fit residual for persons was -.29 (SD = 1.05). The final PSI was .78, indicating adequate person separation reliability. There was no evidence of differential item functioning for sex, age, education level or rural/urban residence.

#### PIPP impact: relationships

**Figure 1:**
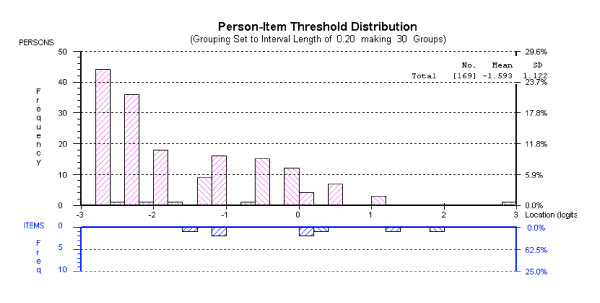
Targeting map for PIPP Impact: Relationship subscale.

Analysis of the four relationship items revealed a non-significant item-trait chi-square statistic (chi-square = 11.90, df = 8, p = .16), indicating adequate fit to the model, with no misfitting items (see Table 2). The mean fit residual value for items was .61(SD = .32), while the mean fit residual for persons was -.23 (SD = 1.13). Items were free of differential item functioning. The Person Separation Index for this subscale was low (.69), due primarily to the fact that there was considerable missing data for the item concerning close relationships. Inspection of the targeting map revealed a floor effect with a clustering of respondents reporting relatively little impact of their health condition on relationships (see Figure 1).

#### PIPP impact: psychological well-being

Rasch analysis of the five items relating to psychological well-being showed good fit to the model (item-trait chi-square = 8.43, df = 10, p = .59) with an adequate PSI value of .73 (see Table 2). The mean fit residual value for items was -.60 (SD = .71), while the mean fit residual for persons was -.74 (SD = 1.4). Inspection of the individual item fit statistics identified no misfitting items or differential item functioning.

### PIPP distress subscales

Consistent with the procedures adopted in the previous section, the items from each of the individual subscales were subjected to Rasch analysis. Disordered thresholds were corrected by recoding all items as 011112.

#### PIPP distress: self-care

The overall model fit statistics for the four Distress Self-care items were non-significant, indicating no serious misfit to the model (item-trait interaction chi-square = 9.42, df = 8, p = .31) (see Table 3). The mean fit residual value for items was .12 (SD = .53), while the mean fit residual for persons was -.29 (SD = .80). The PSI was .79, suggesting adequate person separation reliability, given the small number of items involved. No misfitting items were detected, with all individual item fit statistics being non significant (see Table 3). Items were found to be free of differential item functioning for sex, age, education level and rural/urban residence.

**Table 3:** Individual item fit statistics for PIPP: Distress scale items

	**Item**	**Location**	**SE**	**FitResid**	**DF**	**ChiSq**	**DF**	**Prob**
**Self-care**	4	-0.56	0.23	-0.39	55.18	2.05	2	0.359
	5	-0.04	0.23	0.04	55.18	3.54	2	0.171
	6	-0.70	0.22	0.87	55.18	2.24	2	0.326
	7	1.31	0.27	-0.01	54.46	1.59	2	0.451
								
**Mobility**	9	-0.65	0.17	-0.56	105.56	8.72	2	0.013
	10	-0.30	0.15	-0.25	104.00	0.27	2	0.875
	11	0.01	0.15	0.97	102.44	0.16	2	0.923
	12	1.10	0.17	-0.45	104.78	9.41	2	**0.009**
	13	-0.16	0.15	0.93	103.22	0.50	2	0.780
								
**Participation**	8	-0.09	0.16	2.16	88.60	2.23	2	0.329
	18	0.71	0.17	-0.50	90.87	2.71	2	0.258
	19	0.49	0.17	0.22	89.36	3.87	2	0.144
	20	-0.46	0.15	-0.22	92.38	0.39	2	0.823
	21	-0.65	0.19	-0.64	56.79	5.03	2	0.081
								
**Relationships**	14	0.27	0.18	1.07	55.29	1.27	2	0.531
	15	0.40	0.19	0.28	55.29	1.06	2	0.589
	16	0.12	0.19	0.16	53.17	6.12	2	0.047
	17	-0.79	0.19	0.66	43.24	0.38	2	0.826
								
**Psychological well-being**	1	-0.74	0.19	-0.20	113.86	0.13	2	0.938
	2	0.16	0.18	0.10	113.86	0.26	2	0.877
	3	0.47	0.17	-0.36	113.07	1.26	2	0.532
	22	0.25	0.16	-0.76	108.36	2.45	2	0.295
	23	-0.14	0.15	-1.13	113.86	1.56	2	0.458

Significant values are bolded.

#### PIPP distress: mobility

The five Distress Mobility items showed adequate overall model fit (item-trait interaction chi-square = 19.06, df = 10, p = .04) and good person separation reliability (PSI = .83). The mean fit residual value for items was .13 (SD = .76), while the mean fit residual for persons was -.46 (SD = 1.32). Inspection of the individual item fit statistics (see Table 3) however revealed a misfitting item (item 12: *ability to move around own house*). While removal of this item from the subscale resulted in an improvement in the overall model fit (item-trait interaction chi square = 12.06, df = 8, p = .15), it resulted in a substantial reduction in the PSI, from .83 to .78. It was therefore decided to retain this item in the subscale. There was no evidence of differential item functioning for any of the mobility items.

#### PIPP distress: participation

The five Distress Participation items showed good overall fit to the Rasch (item-trait interaction chi-square = 14.23, df = 10, p = .16) with a PSI of .79 (see Table 3). All thresholds were ordered, there were no misfitting items, and no evidence of differential item functioning. The mean fit residual value for items was .20 (SD = 1.14), while the mean fit residual for persons was -.41 (SD = 1.35).

#### PIPP distress: relationships

Analysis of the four Distress Relationship items revealed a non-significant item-trait chi-square statistic (chi-square = 8.82, df = 8, p = .36) indicating adequate fit to the model, with no misfitting items (see Table 3). The mean fit residual value for items was .54 (SD = .41), while the mean fit residual for persons was -.25 (SD = 1.19). The PSI for this subscale was adequate (.73), however there was considerable missing data for the item concerning close relationships. Items were found to be free of differential item functioning.

#### PIPP distress: psychological well-being

Rasch analysis of the five items relating to psychological well-being showed good fit to the model (item-trait chi-square = 5.66, df = 10, p = .84) with a good PSI value of .83 (see Table 3). The mean fit residual value for items was -.47 (SD = .49), while the mean fit residual for persons was -.76 (SD = 1.55). Inspection of the individual item fit statistics identified no misfitting items or differential item functioning.

### Validation of PIPP subscales

To further assess the characteristics of the PIPP subscales, the person estimates generated in RUMM2020 for each of the subscales were exported to an SPSS data file. These were then subjected to further statistical analyses to assess the relationship among the PIPP subscales, between the impact and distress subscales, and between the subscales and the various components of the EQ5D, which were included in the questionnaire booklet. The descriptive statistics for the Impact and Distress subscales are shown in Table 4. Non-parametric correlation coefficients (Spearman rho) were used given the skewed distribution of scores on a number of the subscales.

**Table 4:** Descriptive statistics for Rasch calibrated scores of PIPP Impact and Distress subscales

		Mean	SD	Min	Max
Impact	Self-care	-2.30	1.78	-4.00	2.65
	Mobility	-0.00	1.74	-4.70	4.23
	Participation	-0.23	1.93	-3.12	3.66
	Relationships	-1.59	1.12	-2.74	2.88
	Psychological well-being	0.17	1.75	-4.73	4.07
					
Distress	Self-care	-2.61	1.51	-3.70	2.47
	Mobility	-0.77	1.86	-3.63	3.70
	Participation	-1.45	1.11	-2.54	2.61
	Relationships	-0.72	1.61	-2.97	3.27
	Psychological well-being	-0.51	2.05	-4.21	3.86

### Intercorrelations among PIPP impact and distress subscales

Table 5 shows the Spearman correlation coefficients (rho) among the PIPP Impact subscales. The strongest correlation was between the impact on Mobility and Psychological Well-being (rho = .61) with the lowest occurring between Self-care and Participation (rho = .206). The correlations among the distress subscales are shown in Table 6. Overall the correlations among the distress subscales were higher than that observed for the Impact items. The highest inter-correlation was between Mobility and Psychological Well-being (rho = .78), with the lowest between Self-care and Relationship (rho = .365).

**Table 5:** Spearman correlation coefficients among PIPP: Impact subscales

Impact subscales	Self-care	Mobility	Participation	Relationships
Self-care				
Mobility	.511			
Participation	.206	.451		
Relationships	.304	.407	.473	
Psychological well-being	.412	.610	.437	.312

All correlations significant at p < .01.

**Table 6:** Spearman correlation coefficients among PIPP: Distress subscales

Distress subscales	Self-care	Mobility	Participation	Relationships
Self-care				
Mobility	.555			
Participation	.396	.646		
Relationships	.365	.482	.558	
Psychological well-being	.505	.784	.670	.483

All correlations significant at p < .01.

Correlation coefficients were also calculated between the corresponding PIPP Impact and PIPP Distress subscales to explore the relationship between respondents' perceptions of the impact of their disability, and the level of distress that this causes (see Table 7). All correlations were above .753 (Mobility), with the highest being recorded for the Relationship subscales (rho = .928).

**Table 7:** Spearman correlations between PIPP: Impact and PIPP: Distress items

	Distress: Self-care	Distress: Mobility	Distress: Particip	Distress: Relation	Distress: Psych
Impact: Self-care	**.827**	.426	.288	.286	.373
Impact: Mobility	.492	**.753**	.484	.385	.600
Impact: Participation	.277	.480	**.841**	.498	.488
Impact: Relationships	.344	.459	.524	**.928**	.443
Impact: Psychological well-being	.433	.612	.502	.290	**.807**

All correlations significant at p < .01.

### Relationship with EQ5D

The validity of the PIPP impact and distress subscales was assessed by investigating the relationship with appropriate corresponding EQ5D items which were included in the questionnaire booklet administered to participants.

#### PIPP psychological well-being

To assess the construct validity of the PIPP Psychological Well-being, subscale scores were compared to those obtained for the EQ5D Anxiety/Depression item. Due to the small number in the extremely anxious/depressed EQ5D response category, respondents were collapsed into two groups: (1) not anxious/depressed (N = 113); and (2) moderately or extremely anxious/depressed (N = 56). Mann-Whitney tests were conducted to compare the scores for the two groups on the PIPP Impact and PIPP Distress Psychological Well-being subscales. There was a highly statistically significant difference between respondents in the two EQ5D groups for both the PIPP Psychological Well-being Impact subscale (z = -3.967, p < .001) and the PIPP Psychological Well-being Distress subscale (z = -5.30, p < .001). Respondents classified as moderately or extremely anxious/depressed on the EQ5D recorded higher mean rank scores than the not anxious/depressed group on each of the PIPP Psychological Well-being subscales (Impact: 105.84 vs 74.67; Distress: 113.04 vs 71.10), supporting the validity of these subscales.

#### PIPP self-care

The PIPP Self-care subscales were compared with the EQ5D item concerning self-care. Due to the small numbers of respondents in the 'unable' response category of the EQ5D Self-care item respondents were collapsed into two categories: (1) no problems (N = 96), and (2) some problems or unable to care for self (N = 73). Mann-Whitney tests revealed significant differences between the two groups on the PIPP Self-care Impact (z = -6.789, p < .001) and PIPP Self-care Distress subscales (z = -5.106, p < .001). The mean rank scores on each PIPP subscale was substantially higher for the respondents classified as having self-care problems on the EQ5D (Impact: 113.05 vs 63.67; Distress: 105.10 vs 69.17), supporting the validity of the self-care PIPP subscales.

#### PIPP mobility

Mann-Whitney tests were conducted to compare the PIPP Mobility subscale scores for the collapsed response categories of the EQ5D Mobility item (no problems vs some problems/confined to bed). There was a statistically significant difference for both the PIPP Mobility Impact (z = -4.092, p < .001) and Distress (z = -2.733, p = .006) subscales. There was a difference, in the expected direction, of the mean ranks for respondents reporting no problems vs the respondents with mobility problems (Impact: 9.28 vs 40.69; Distress: 88.53 vs 55.39).

#### PIPP participation

Kruskal-Wallis tests were conducted to compare PIPP Participation scores for respondents in each of the three response categories to the EQ5D item 'Usual Activities' (no problems, some problems, unable to perform). There was a statistically significant difference in scores on the PIPP Impact (chi-square = 16.53, df = 2, p = .001) and PIPP Distress (chi-square = 23.31, df = 2, p = .001) subscales. Mean ranks for each group were in the expected direction with the 'unable to perform' groups showing the highest PIPP Participation scores (Impact: 106.56 vs 87.64 vs 59.96; Distress: 105.70 vs 90.58 vs 53.04)

#### EQ5D VAS

In addition to the individual domain items, the EQ5D VAS asks respondents to rate their health in general today on a scale from 0 to 100. The correlations between this rating and each of the PIPP Impact and Distress subscales Rasch calibrated scores are shown in Table 8. Correlations ranged from -.20 (Impact Relationship and Distress Relationships) to -.388 (Impact Psychological Well-being).

**Table 8:** Spearman correlations between PIPP scales and EQ5D 'Health in general today' item

		Health in general today
Impact	Self-care	-.297 **
	Mobility	-.366 **
	Participation	-.303 **
	Relationships	-.200*
	Psychological well-being	-.388 **
		
Distress	Self-care	-.289 **
	Mobility	-.348 **
	Participation	-.358 **
	Relationships	-.200 *
	Psychological well-being	-.381 **

*p < .01. **p < .001.

## Discussion

The aim of this study was to develop, and conduct preliminary validation, of a multidimensional generic measure of the impact and distress of health conditions from the individual's perspective. For both the Impact and Distress domains of the PIPP, the five subscales (Self-care, Mobility, Participation, Relationships, Psychological Well-being) showed adequate psychometric properties, demonstrating fit to the Rasch model, adequate person separation reliability and no evidence of differential item functioning.

Initially it was necessary to collapse the original 6-point response scale to a simpler 3-point response scale to resolve disordered thresholds. The presence of disordered thresholds suggests that the respondents were unable to reliably differentiate the original six response points. Before recommending a change to the response format of the PIPP, this finding requires further investigation in other studies across different health conditions, and different cultural contexts. The simpler 3-point response scale (which is commonly used in other health scales, such as the EQ5D [15]) may have the advantage of reducing the cognitive complexity of the task of completing the PIPP ratings. In this case the use of three response points could be labelled: *no impact/distress*, *moderate impact/distress *and *extreme impact*/*distress*.

In order to achieve satisfactory fit to the Rasch model it was necessary to remove one item of the PIPP Impact: Participation scale (item 8: *assist other family members*). Removal of this item improved the fit statistics of the subscale, indicating that it was not appropriate to retain this item in the subscale in the current study. It is interesting to note that there was no evidence of misfit in the corresponding item in the PIPP Distress: Participation scale. Given the relatively small sample involved in this study the generalisability of this finding should be further investigated in larger studies, involving different health conditions.

The results of this study indicated a floor effect in regard to the Relationship subscale, with a clustering of respondents recording low scores suggesting that their health condition had little impact on their relationships. However, the correlation between impact and distress subscales for relationships was the highest among all domains (rho = 0.928). This suggests that while relationships are comparatively more resistant to the impact of mobility impairment then other domains, when a health condition does actually impact upon relationships, it results in considerable distress. The importance of relationships in terms of rehabilitation and coping with a health condition has been well documented in the literature.

One of the major strengths of the PIPP is that it measures both impact and distress in regard to a variety of domains, providing valuable information on the individual's experience of their health condition. Overall, the correlations for the subscales were stronger between the distress items, as compared to the impact items. Among the impact subscales, the strongest association was between mobility and psychological well-being, and mobility and self-care. Among the distress subscales, the strongest correlations were between mobility, participation, and psychological well-being. The relationship between mobility and the other subscales for both impact and distress indicate the pervasive impact of mobility impairment, and supports its inclusion into ICF core sets for a range of different health conditions [12].

Correlations between the impact and distress scores for each of the subscales ranged from .75 to .93, indicating substantial overlap between these two aspects. Exploration of the dimensionality of the set of PIPP subscales is needed, including an assessment of the appropriateness of combining subscales to provide a simpler global score for use in research contexts. Further research is also needed to assess the potential use of the detailed profile of PIPP impact and distress scales in clinical contexts.

Preliminary validity testing using the EQ5D items and VAS health rating showed support for both the PIPP Impact and Distress subscales. While the EQ5D measures how much difficulty a person has in relation to various domains, the PIPP measures the subsequent impact and distress related to similar domains. In this study, those who indicated higher levels of anxiety/depression on the EQ5D were more likely to report higher impact and distress in regard to their psychological well-being. Similarly, those who had more difficulty in regard to self-care, mobility, and participation as measured by EQ5D, were more likely to also report higher impact and distress in the equivalent PIPP subscales.

The ICF was used in this study to guide the conceptualization and structure of the PIPP with the inclusion of subscales representing many of the ICF elements that have been proposed as core domains for a wide spectrum of serious health conditions. While the ICF focuses on the classification of objective health and health related status, the PIPP broadens this focus to address the subjective experience of health from the individuals' perspective [23]. In addition to the ICF elements of self-care, mobility, participation and relationships, the PIPP also incorporated items relating to psychological well-being. While these aspects are not included in the current ICF framework, these areas have been marked by the WHO for further development with the intention of 'establishing links with quality of life concepts and the measurement of subjective well-being'[1].

Consistent with the biopsychosocial model, the PIPP provides an integrated tool with items tapping the physical, social and psychological impact and distress of a health condition from the individual's perspective. It is designed to supplement existing objective measures of functioning that focus on the individual's ability to perform certain tasks or activities. As a generic measure, rather than disease specific, it is potentially useful (subject to further psychometric assessment) for use across a range of health conditions.

In this initial validation study the focus has been on the impact and distress experienced by individuals with mobility impairments. Further research is necessary to test the applicability of the PIPP across different health conditions and in different cultural contexts. While this study was conducted on a relatively small Australian sample, it forms part of a larger cross-cultural study that explores the impact and distress related to mobility in different countries, including Thailand, Malaysia, Myanmar, and Laos. Therefore, further validation of PIPP will be conducted within and across the different countries. Further investigation is also warranted concerning the potential use of the PIPP in clinical and rehabilitation settings. It may provide clinicians with a measurement tool supplying valuable information concerning the experience of the individual client, guiding the planning of therapeutic interventions and assessment of treatment outcome. Copies of the PIPP may be obtained free of charge for non-commercial use from the corresponding author.

## Competing interests

The author(s) declare that they have no competing interests.

## Authors' contributions

JP, EB and LM designed the study, and obtained the data. JP and RM undertook the literature review and conducted the statistical analyses. All authors contributed to the preparation of the article and approved the final manuscript.
